# Sex and education differences in trajectories of physiological ageing: longitudinal analysis of a prospective English cohort study

**DOI:** 10.1093/ageing/afaf067

**Published:** 2025-03-29

**Authors:** Mikaela Bloomberg, Andrew Steptoe

**Affiliations:** Department of Epidemiology and Public Health, University College London, 1-19 Torrington Place, London, Greater London WC1E 7HB, UK; Department of Behavioural Science and Health, University College London, 1-19 Torrington Place, London, Greater London WC1E 7HB, UK

**Keywords:** sex differences, phenotypic ageing, physiological ageing, socioeconomic inequalities, biological ageing, older people

## Abstract

**Background:**

Physiological age (PA) derived from clinical indicators including blood-based biomarkers and tests of physiological function can be compared with chronological age to examine disparities in health between older adults of the same age. Though education interacts with sex to lead to inequalities in healthy ageing, their combined influence on longitudinally measured PA has not been explored. We derived PA based on longitudinally measured clinical indicators and examined how sex and education interact to inform PA trajectories.

**Methods:**

Three waves of clinical indicators (2004/05–2012/13) drawn from the English Longitudinal Study of Ageing (ages 50–100 years) were used to estimate PA, which was internally validated by confirming associations with incident chronic conditions, functional limitations and memory impairment after adjustment for chronological age and sex. Joint models were used to construct PA trajectories in 8891 English Longitudinal Study of Ageing participants to examine sex and educational disparities in PA.

**Findings:**

Amongst the least educated participants, there were negligible sex differences in PA until age 60 (sex difference [men–women] age 50 = −0.6 years [95% confidence interval = −2.2 to 0.6]; age 60 = 0.4 [−0.6 to 1.4]); at age 70, women were 1.5 years (0.7–2.2) older than men. Amongst the most educated participants, women were 3.8 years (1.6–6.0) younger than men at age 50 and 2.7 years (0.4–5.0) younger at age 60, with a nonsignificant sex difference at age 70.

**Interpretation:**

Higher education provides a larger midlife buffer to physiological ageing for women than men. Policies to promote gender equity in higher education may contribute to improving women’s health across a range of ageing-related outcomes.

## Key Points

- Women generally have higher physiological ages than men.
- Education above high school level provides a larger midlife benefit to physiological age for women than it does for men.
- Sex disparities in physiological ageing are attenuated in highly educated women.

## Introduction

It is well documented that the central ageing processes driving ageing-related pathophysiological changes are not perfectly correlated with chronological age (CA) [1, 2], which is one factor leading to considerable heterogeneity in health between older adults of the same age. Biological ageing metrics based on telomere length or DNA methylation aim to explain variability in ageing-related disease better than CA alone [3–6]. However, biological age derived from clinical indicators including blood-based biomarkers and tests of physiological function [6–11]—sometimes referred to as physiological or phenotypic age—may explain variation in health outcomes better than molecular or cellular measures because these indicators reflect physiological changes more closely related to health outcomes [7, 9]. Where epigenetic or telomere-based biological clocks measure aspects of the central ageing process, physiological age captures the downstream effects of the ageing process on multiple organ systems [9]. We can then identify characteristics that predict physiological age younger or older than CA and thus use physiological age to examine disparities in healthy ageing.

Socioeconomic inequalities are a major contributor to disparities in healthy ageing [12]. Amongst socioeconomic characteristics, education is of particular importance due to its propagating effects on socioeconomic position during the life course, influencing a broad range of health-related factors including access to healthcare, health behaviours and health literacy and culminating in its impact on late-life morbidity and mortality [13, 14]. Education also has key interactions with gender—which is commonly informed by sex—as historically, women were restricted in access to education and limited to lower education levels, though gender differences in education may be progressively reduced in later birth cohorts [15]. Whilst there is a substantial body of literature examining how interactions of sex and education impact individual health outcomes, and independent associations of sex and education with biological age, how sex and education combine to influence physiological ageing is not yet clear. Previous studies suggest lower biological ages in more educated individuals [16–18] and women [19–24], whilst studies examining sex differences using longitudinally measured physiological age undertaken in small, highly selected study populations (*N* = 179–802) generally suggest lower physiological ages for men [25–27].

To examine how sex and education combine to inform physiological ageing, we derived a physiological ageing measure based on clinical indicators assessed at three time points (2004/05, 2008/09 and 2012/13). We examined sex and educational differences in longitudinal physiological ageing trajectories from 8891 participants aged 50–100 years from the English Longitudinal Study of Ageing (ELSA).

## Methods

### Data sources

ELSA is a nationally representative cohort study of the English population aged ≥50 years with biennial data collection beginning in 2002/03 and continuing until 2018/19; the most recent wave of data collection occurred in 2021/23. Details of the survey design are available elsewhere [28]. Data were drawn from Waves 2 (2004/05), 4 (2008/09) and 6 (2012/13), during which ELSA participants underwent a series of physiological assessments and had blood samples drawn in a home visit from a study nurse. ELSA participants with at least one nurse visit during these waves were eligible for inclusion in analyses. We did not include data from further nurse visits at Waves 8 and 9 because lung function was not assessed.

### Sex, education and covariates

At the first wave of biomarker collection, participants were asked to report their ‘sex’ as ‘male’ or ‘female’. ELSA survey materials do not delineate between sex and gender; sex was referred to in the survey and is therefore used in the present study. Participants were also asked about their highest educational qualification: less than high school (low education), high school (intermediate education) or above high school (high education).

Participants were asked to report their birth year, which was categorised into 7-year birth cohorts (1911–62), chosen to maximise the age range in each birth cohort whilst minimising birth cohort effects and allowing birth cohorts to capture major sociohistorical events (e.g. World War II).

### Derivation and internal validation of physiological age

Clinical indicators collected during nurse visits included those pertaining to the cardiovascular system (pulse pressure, systolic blood pressure, diastolic blood pressure, mean arterial pressure), the respiratory system (forced vital capacity [FVC], forced expiratory volume in 1 second [FEV]), the haematologic system [haemoglobin concentration, fibrinogen, C-reactive protein (CRP), ferritin], metabolism (fasting glucose, glycated haemoglobin, total cholesterol, low-density lipoprotein cholesterol, high-density lipoprotein cholesterol, triglyceride) and the musculoskeletal system (grip strength, waist circumference). Procedures for indicator collection and processing are reported in Appendix 1 (Methods 1a).

Derivation of physiological age was performed using the principal component analysis method, a commonly used method of physiological age estimation. [29–32]; see Appendix 1 (Methods 1b) for details. Clinical indicators selected for inclusion in physiological age were pulse pressure, systolic blood pressure, fibrinogen, CRP, glycated haemoglobin, FEV, FVC and grip strength (Appendix 3, Table 3a). The correlation between physiological age and CA was 0.76–0.78 for all waves (Appendix 3, Table 3b). We estimated ageing acceleration by subtracting CA from physiological age, where a positive value for ageing acceleration indicated accelerated ageing, as has been done in previous studies [29–32].

To internally validate physiological age as a healthy ageing index, we used Cox proportional hazards models adjusted for CA and sex to examine associations between each individual’s first measurement of ageing acceleration and incidence of the following ageing-related outcomes occurring during Waves 2–10 (2004/05–2021/23) of ELSA (Appendix 2, Figure 2a): (i) functional limitations in activities of daily living and mobility activities; (ii) memory impairment; and (iii) ageing-related chronic conditions. Accelerated ageing was associated with an increased risk of all outcomes examined except cancer and Parkinson’s disease. Details are available in Appendix 1 (Methods 1c).

### Trajectories of physiological age

We used joint models to examine trajectories of physiological age. These models simultaneously estimate a longitudinal submodel (linear mixed model with a CA time scale and a random intercept and slope at the individual level) and a survival submodel (Weibull model), which are linked through shared random effects and can be used to account for data missing-not-at-random in the longitudinal process [33]. In the present study, individuals with older physiological ages are more likely to be of poor health and are therefore more likely to die or be lost to follow-up. Consequently, results from linear mixed models—which account for data missing-at-random—would be biased [34], leading us to use joint models, which take attrition into account in the estimation of the longitudinal model.

Model 1 included CA centred at age 50*,* birth cohort and the interaction between birth cohort and CA (denoted ‘birth cohort × CA’) to allow trajectories of physiological age to differ between birth cohorts*.* Visual inspection using local polynomial regression suggested the relationship between physiological age and CA was linear (Appendix 2, Figure 2b), leading us to exclude higher-order CA terms. After first examining including birth cohort in the models as a categorical variable, birth cohort was ultimately included as a continuous variable centred on the 1939–45 cohort to improve model parsimony. To examine sex differences in physiological ageing, we added sex and sex × CA to Model 1 to yield Model 2. For Model 3, we added education and education × CA to Model 2 to examine educational differences in physiological ageing. Finally, for Model 4, we added sex × education and sex × education × CA to Model 3 to determine whether sex differences in physiological age differed by education level.

We used these models to plot trajectories of physiological age from chronological ages 50–80 years in men and women (Model 2), by education level (Model 3) and in men and women by education level (Model 4). This age range was chosen to reduce birth cohort effects. We reported sex and education differences in physiological age at chronological ages 50, 60 and 70 and differences in the rate of physiological ageing. Analyses were performed in StataMP 18.0 or R 4.2.2 with a two-sided *P* < .05 considered significant.

### Additional analyses

We re-ran Model 2 including interactions between sex, birth cohort and CA and Model 3 including interactions between education, birth cohort and CA to determine whether sex or educational differences in physiological ageing differed across birth cohorts.

## Results

### Sample characteristics

Of 12 291 ELSA respondents aged ≥50 years with at least one nurse visit during waves 2, 4 or 6, 8906 (72.5%) had complete biomarker data. Of these 8906, 15 (0.2%) were missing education and were excluded, leading to 8891 participants aged 50–99 years at enrolment retained for the analyses (Appendix 2, Figure 2c). Excluded respondents were similar with respect to age and gender, though slightly less educated than included respondents (Appendix 3, Table 3c). Table 1 shows sample characteristics. Of 8891 participants, 4094 (46.0%) were men and 4797 (54.0%) were women. Men and women had the same mean chronological age (64.1 years, SD = 9.1 for men or 9.4 for women; *P* = .94). Men were more educated than women (*P* < .0001). The mean follow-up duration was the same for men and women (mean = 2.6 years [SD = 3.1]).

**Table 1:** Characteristics of the analytic sample at first physiological age measurement

	Men *N* = 4094	Women *N* = 4797	*P*-value
Chronological age, mean (SD)	64.1 (9.1)	64.1 (9.4)	.94
Physiological age, mean (SD)	68.4 (18.7)	69.2 (20.4)	.045
Ageing acceleration, mean (SD)	4.2 (13.1)	5.1 (14.3)	.0048
Highest educational qualification			
Less than high school	1586 (38.7)	2198 (45.8)	<.0001
High school	1849 (45.2)	2107 (43.9)	
Above high school	659 (16.1)	492 (10.3)	

*N* (%) shown unless otherwise indicated. Abbreviation: SD, standard deviation.

At first physiological age measurement, men had younger physiological ages than women (mean = 68.4 years [SD = 18.7] for men, mean = 69.2 [SD = 20.4] for women; *P* = .045), with physiological ages on average 4.2 years (SD = 13.1) older than their chronological age compared with 5.1 years (SD = 14.3) for women (*P* = .0048). Participants in the low education group had physiological ages on average 9.3 years (SD = 13.7) older than their chronological age or 2.1 years (SD = 12.8) older in the intermediate education group. Participants in the high education group had physiological ages on average 1.7 years (SD = 12.3) younger than their chronological age.

### Trajectories of physiological age

Table 2 shows coefficients from physiological age models. After adjusting for the birth cohort, participants had an average physiological age of 49.4 years (48.7–50.2) at chronological age 50 years, 63.2 years (62.8–63.6) at age 60 and 77.0 years (76.6–77.4) at age 70. Physiological age increased by 1.4 years (1.3–1.4) per year increase in chronological age.

**Table 2:** Coefficients from models for physiological age

Model	Coefficient (95% CI)	*P*-value
(1) Overall		
Intercept	49.4 (48.7 to 50.2)	<.0001
CA	1.4 (1.3 to 1.4)	<.0001
(2) Sex (ref. male)		
Female	−0.9 (−1.8 to −0.0)	.047
Female × CA	0.1 (0.1 to 0.2)	<.0001
(3) Education (ref. low)		
Intermediate	−4.7 (−5.7 to −3.7)	<.0001
High	−7.6 (−9.0 to −6.2)	<.0001
Intermediate × CA	0.1 (−0.0 to 0.1)	.075
High × CA	0.0 (−0.1 to 0.1)	.45
(4) Sex and education		
Female	−0.6 (−2.2 to 0.9)	.42
Female × CA	0.1 (0.03 to 0.2)	.0039
Intermediate	−4.5 (−6.0 to −2.9)	<.0001
High	−6.0 (−7.9 to −4.1)	<.0001
Intermediate × CA	0.0 (−0.0 to 0.1)	.25
High × CA	−0.0 (−0.1 to 0.1)	.86
Female × intermediate	−0.4 (−2.4 to 1.6)	.68
Female × high	−3.2 (−5.8 to −0.5)	.021
Female × intermediate × CA	0.0 (−0.1 to 0.1)	.97
Female × high × CA	0.1 (−0.1 to 0.2)	.37

Unit for chronological age and physiological age is years. Models are centred at chronological age 50; the intercept for Model 1 therefore corresponds to the mean physiological age at chronological age 50 in the sample for the reference birth cohort (1939–45). All models also include birth cohort, the interaction between birth cohort and chronological age and all terms from the previous model (e.g. Model 2 includes all Model 1 terms) with interaction terms denoted using ‘×’. Model coefficients for Models 2–4 are interpreted as difference in physiological age at chronological age 50 or change in difference in physiological age per year increase in chronological age, e.g. ‘Female’ term from Model 2 is the sex difference [female–male] in physiological age at chronological age 50 and ‘Female × CA’ corresponds to the change in sex difference in physiological age per one year increase in chronological age. Abbreviations: CI, confidence interval; Ref, reference; CA, chronological age.

**Figure 1:**
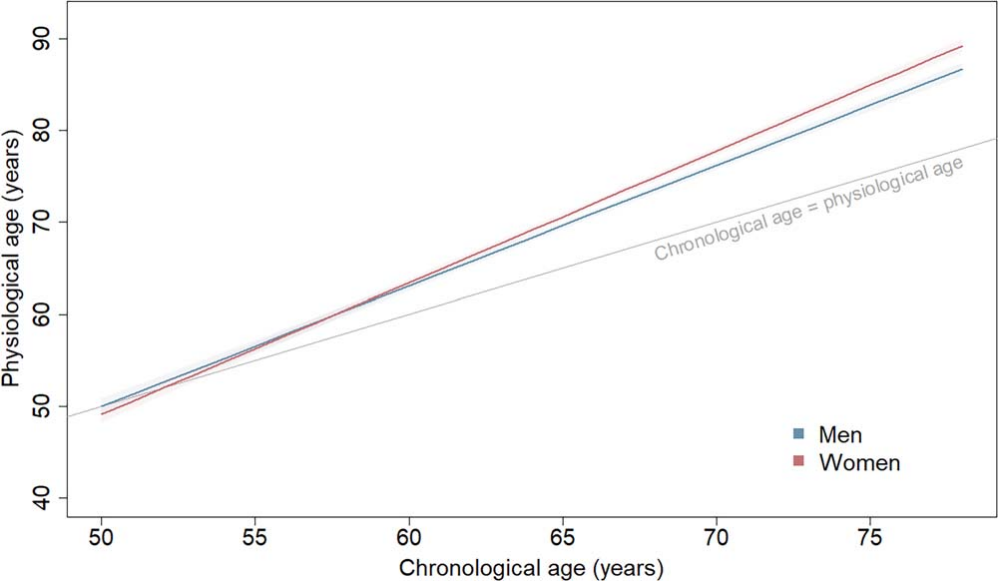
Physiological ageing trajectories from age 50 to 80 years in men and women. Based on the model including chronological age, birth cohort, sex and interactions of: (1) birth cohort and chronological age; and (2) sex and chronological age. Plotted for reference values of covariates (1939–45 birth cohort).

At age 50, women were 0.9 years (0.0–1.8) years younger than men (Figure 1). However, the physiological ages of women increased faster than men (*P*_sex × CA_ < .0001); with each year increase in chronological age, the sex difference in physiological age increased by 0.1 years (0.1–0.2). As a result, women had similar physiological ages to men at age 60 (0.3 years [−0.2 to 0.9] older than men), but by age 70 were 1.6 years (1.0–2.1) older than men.

**Figure 2:**
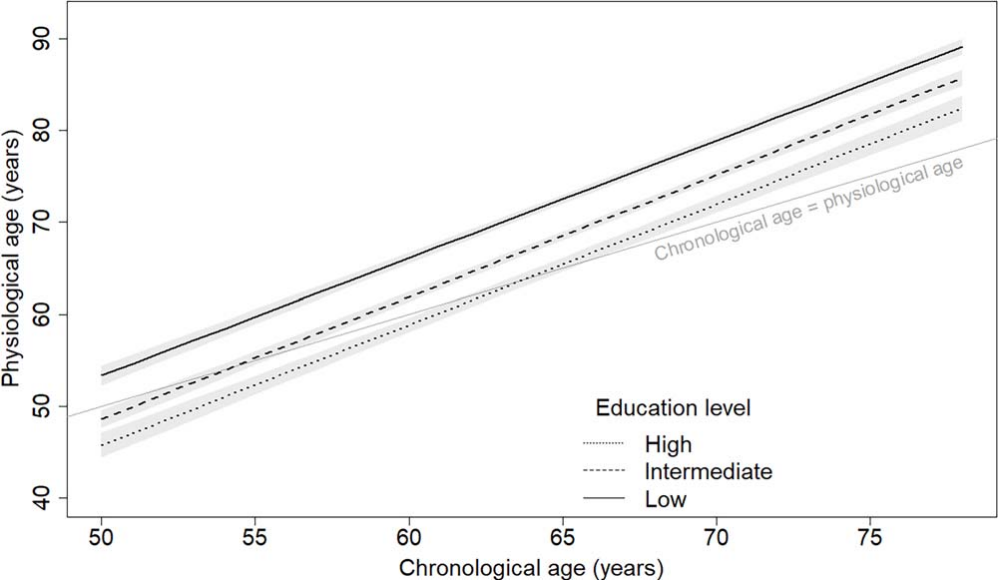
Physiological ageing trajectories from age 50 to 80 years by education level (low = below high school; intermediate = high school; high = above high school). Based on the model including chronological age, birth cohort, sex, education and interactions of: (1) birth cohort and chronological age; (2) sex and chronological age; and (3) education and chronological age. Plotted for reference values of covariates (men, 1939–45 birth cohort).

After adjusting for birth cohort and sex, participants in the intermediate and high education groups respectively had physiological ages 4.7 years (3.7–5.7) and 7.6 years (6.2–9.0) younger than the low education group (Figure 2). There were negligible differences in the pace of physiological ageing between education categories (*P*_intermediate education × CA_ = .075; *P*_high education × CA_ = .45). As a result, education differences in physiological ageing remained mostly consistent. At age 60, participants in the intermediate education group had physiological ages 4.2 years (3.6–4.9) younger than the low education group or 3.7 years (3.2–4.3) younger at age 70. The corresponding values for the high education group were 7.3 years (6.4–8.1) younger than the low education group at age 60 or 6.9 years (6.0–7.9) younger at age 70.

**Figure 3:**
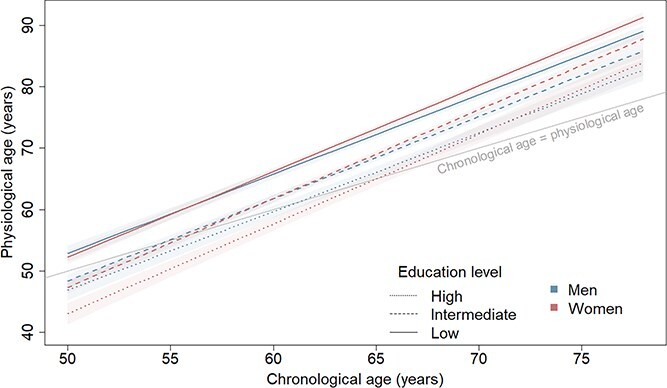
Physiological ageing trajectories from age 50 to 80 years in men and women by education level (low = below high school; intermediate = high school; high = above high school). Based on the model including chronological age, birth cohort, sex, education and interactions of: (1) birth cohort and chronological age; (2) sex and chronological age; (3) education and chronological age; (4) sex and education; and (5) sex, education and chronological age. Plotted for reference values of covariates (1939–45 birth cohort).

In general, sex differences in physiological ageing were similar for low and intermediate education levels (*P*_intermediate education × sex_ = 0.68; Figure 3) and sex differences in the pace of physiological ageing did not vary across levels of education (*P*_intermediate education × sex × CA_ = 0.97; *P*_high education × sex × CA_ = 0.37). However, sex differences in physiological age in the high education group differed from the low education group (*P*_high education × sex_ = 0.021). In the low education group, women had similar physiological ages to men at age 50 (0.6 years [−0.9 to 2.2] younger than men) and 60 (0.4 years [−0.6 to 1.4] older than men) and were 1.5 years (0.7–2.2) older than men at age 70 years; sex differences were similar to the low education group in the intermediate education group. By contrast, in the high education group, women had physiological ages 3.8 years (1.6–6.0) younger than men at age 50, 2.7 years (0.4–5.0) younger than men at age 60 and 1.7 (−0.9 to 4.3) years younger than men at age 70, though the sex difference at age 70 did not reach statistical significance.

### Additional analyses

Examination of models including interactions between sex, education, birth cohort and chronological age suggested that results were similar for all birth cohorts (Appendix 3, Table 3d).

## Discussion

After deriving a physiological ageing measure and examining physiological ageing trajectories in 8891 adults aged 50–100 years, we found key sex and educational differences in physiological ageing. First, before taking education into account, sex differences in physiological age were minor before chronological age 60 but grew progressively larger as women aged faster than men. Second, more education was associated with lower physiological age but no difference in the pace of physiological ageing. Finally, examination of interactions between sex and education revealed that high education provided a midlife benefit for women, such that women educated above high school level were physiologically younger than men until chronological age 70. By contrast, women educated to high school level or below had physiological ages similar to men until chronological age 60 and physiological age increasingly older than men from age 60 onwards. These results suggest education above high school level may be important to reduce female disadvantages in physiological ageing.

The main strengths of this study include its longitudinal measure of physiological age, broad age and birth year range and data drawn from a large and nationally representative study population. Previous cross-sectional studies examining sex and educational disparities in physiological ageing may be influenced by birth cohort effects, wherein systemic improvements in factors such as socioeconomic conditions and healthcare lead to overestimating physiological age at older chronological ages. The broad age range—where ages are represented in multiple birth cohorts—allowed us to consider birth cohort effects in our analysis. Our physiological age was associated with a range of ageing-related health outcomes pertaining to multiple organ systems, indicating that it is an internally valid summary measure of ageing. Finally, we accounted for differential attrition in the analytic sample by simultaneously modelling attrition using joint models.

This study has several limitations. We were limited in the choice of clinical indicators to those routinely collected during nurse visits in ELSA. We were unable to externally validate our measure of physiological ageing because comparable longitudinally measured clinical indicators are not yet widely available in other similar national cohort studies of ageing, so we emphasise that the physiological ageing measure produced in the present study is intended as an internally validated healthy ageing index. Though sex hormones and other reproductive factors may contribute to sex differences in physiological ageing, these measures were not available in ELSA for inclusion in physiological age estimation. Finally, due to missing data, we could not account for height/weight differences in grip strength, though we standardised all clinical indicators by sex, which should have accounted for major biological differences in grip strength.

Whilst previous findings using epigenetic clocks suggest minor sex differences or lower biological ages in women [19–24], our results are consistent with studies using clinical-indicator-based physiological age, which generally suggest that women have higher physiological ages than men [25–27]. This evidence supports the adage that women ‘live longer in worse health’ than men, where lower epigenetic age corresponds to greater longevity and older physiological ages are consistent with experiencing worse health. We also found that women aged faster than men. Previous studies examining sex differences in physiological ageing estimated from longitudinally measured clinical indicators give mixed results [25–27]. One study was undertaken in 179 healthy Japanese adults aged 30–77 years at baseline and found that women aged slightly slower than men but had higher physiological ages at baseline [25]; two studies were undertaken in up to 802 participants aged 45–85 at baseline from the Swedish Adoption/Twin Study of Aging to find negligible sex differences in the pace of physiological ageing but higher physiological ages in women [26, 27]. Our study expands on this previous evidence based on highly selected study populations, as it examines physiological ageing using data drawn from a large, nationally representative study that includes participants reporting chronic conditions.

Health benefits of education include access to healthcare, improved health literacy and engagement in healthy behaviours [35]. Expanding on previous results demonstrating accelerated biological ageing in less educated individuals [16–18], we showed that education above high school level afforded a larger benefit to physiological age for women compared with men. This is consistent with the theory that education may confer greater health benefits for women because women otherwise have fewer socioeconomic resources such as power, authority or earnings [36]. Furthermore, whilst more education is associated with less engagement in unhealthy behaviours for both men and women [37], highly educated men are still more likely to engage in risky behaviours and may be less likely to institute healthy behaviour changes than highly educated women [38].

As younger physiological age relative to chronological age in the present study is associated with risk reduction for a range of adverse ageing-related health outcomes, these results highlight how policies to improve gender equity in higher education might reduce gender disparities in healthy ageing by providing women with a midlife buffer to physiological decline. Whilst the gender gap in higher education has been reduced substantially or even reversed in many high-income countries [39], low- and middle-income countries still see considerable gender disparities in education level, partly due to the persistent societal expectation that women should prioritise homemaking [40]; low- and middle-income settings remain a critical focus for policies aimed at promoting gender equity. This study also provides avenues for future research, including cross-cohort comparisons to assess the role of social determinants of health in disparities in physiological ageing in different settings and evaluate the generalisability of the results. Future studies should also explore mechanisms underlying sex and educational differences in physiological ageing with consideration of secular changes in gender inequalities in education.

It is well established that education has propagating effects on health during the life course [14]. The present results highlight higher education as particularly important for improving trajectories of physiological ageing for women. As the gender gap in higher education is progressively closed, whether women will continue to ‘live longer in worse health’ remains to be seen.

## Supplementary Material

AA-24-1386_Appendix_1_afaf067

AA-24-1386_Appendix_2_afaf067

AA-24-1386_Appendix_3_afaf067

## Research Data Transparency and Availability:

ELSA data are available to researchers after registration with the UK data service at https://beta.ukdataservice.ac.uk/datacatalogue/series/series?id=200011.
