# Supplementary material for: Sex and education differences in trajectories of physiological ageing: longitudinal analysis of a prospective English cohort study
Source: Age Ageing. 2025 Mar 29;54(4):afaf067. doi: 10.1093/ageing/afaf067 (PMC11954548; doi:10.1093/ageing/afaf067)

Sex and education differences in trajectories of physiological ageing: longitudinal analysis of a prospective English cohort study

Appendix 2. Supplemental figures

Figure 2a. Study design.

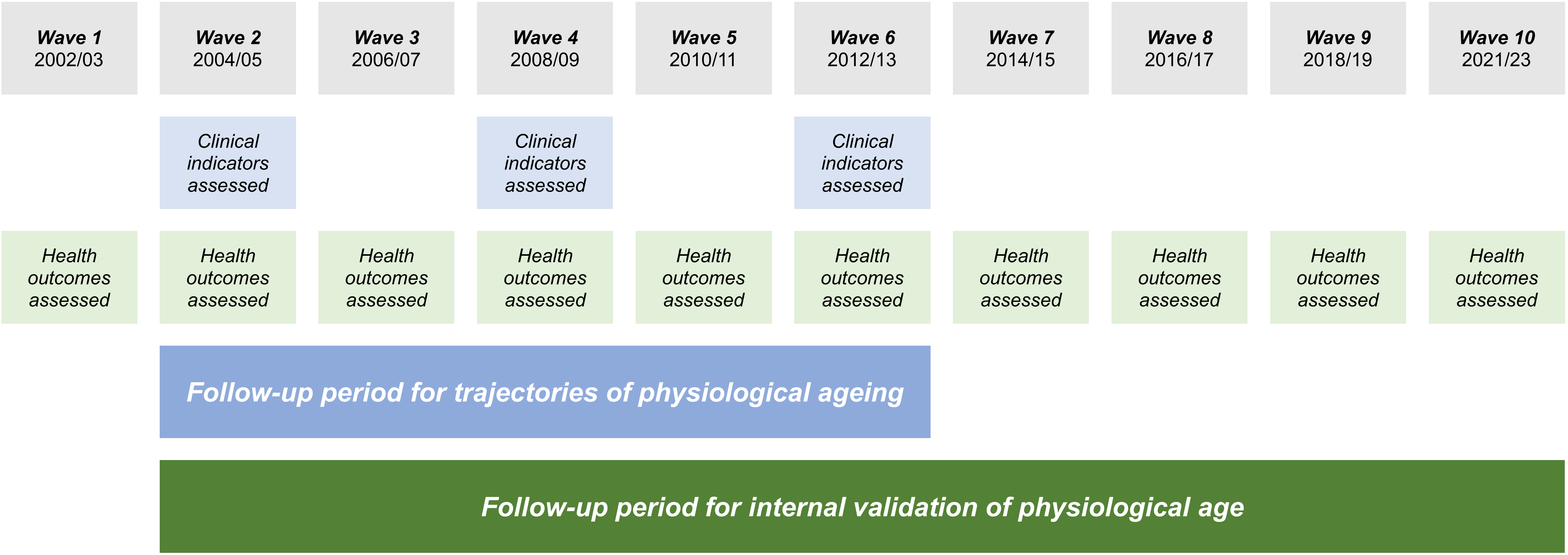

'Clinical indicators' are used in the derivation of physiological age. 'Health outcomes' refer to ageing-related outcomes assessed as part of validation of physiological age: limitations in activities of daily living, instrumental activities of daily living, and mobility limitations, memory impairment, and ageing-related chronic conditions (diabetes, lung disease, cardiovascular disease, stroke, high cholesterol, high blood pressure, cancer, arthritis, osteoporosis, dementia, or Parkinson's disease).

Figure 2b. Physiological age plotted against chronological age in the analytic sample using local polynomial regression.

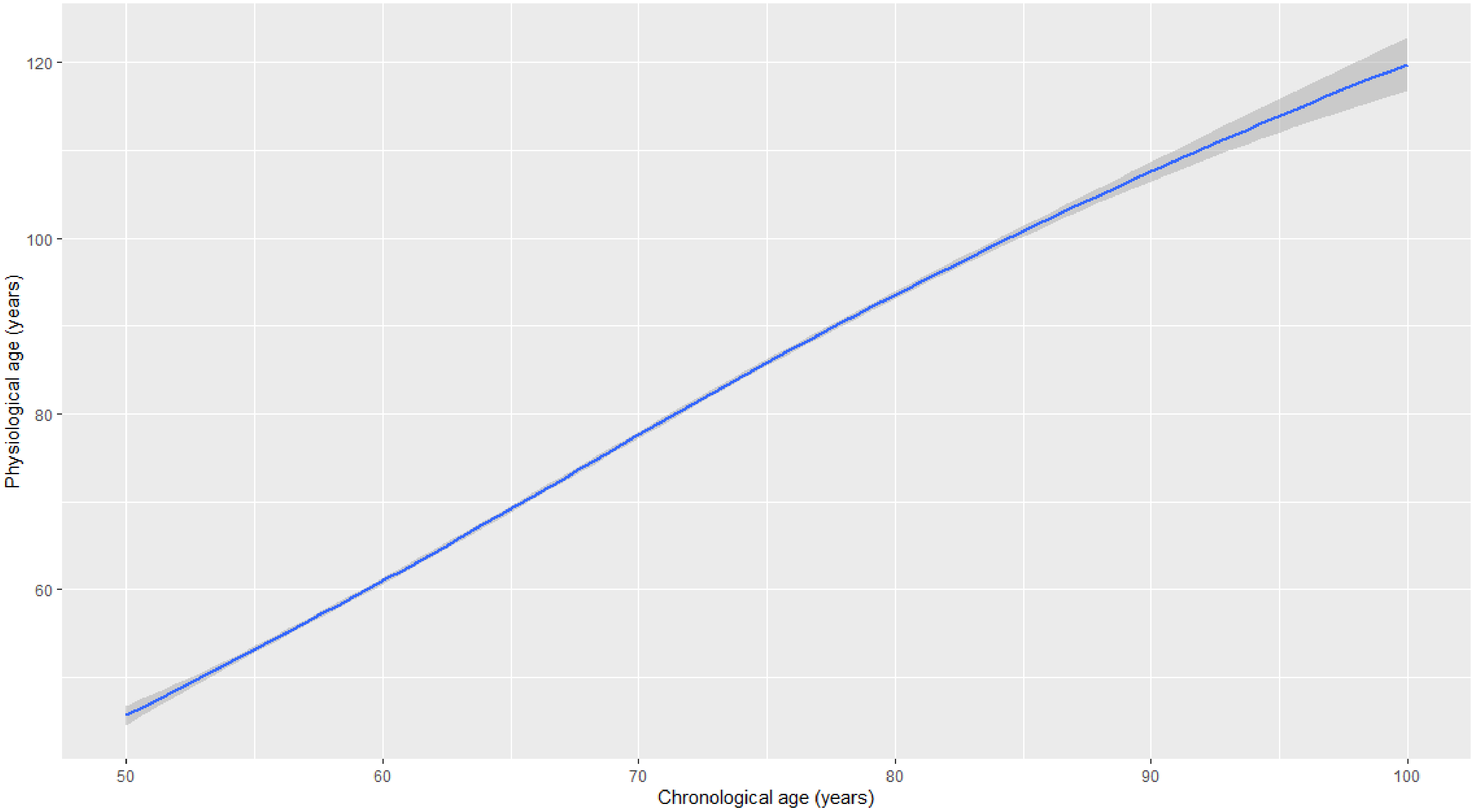

Figure 2c. Flowchart of sample selection for main analysis.

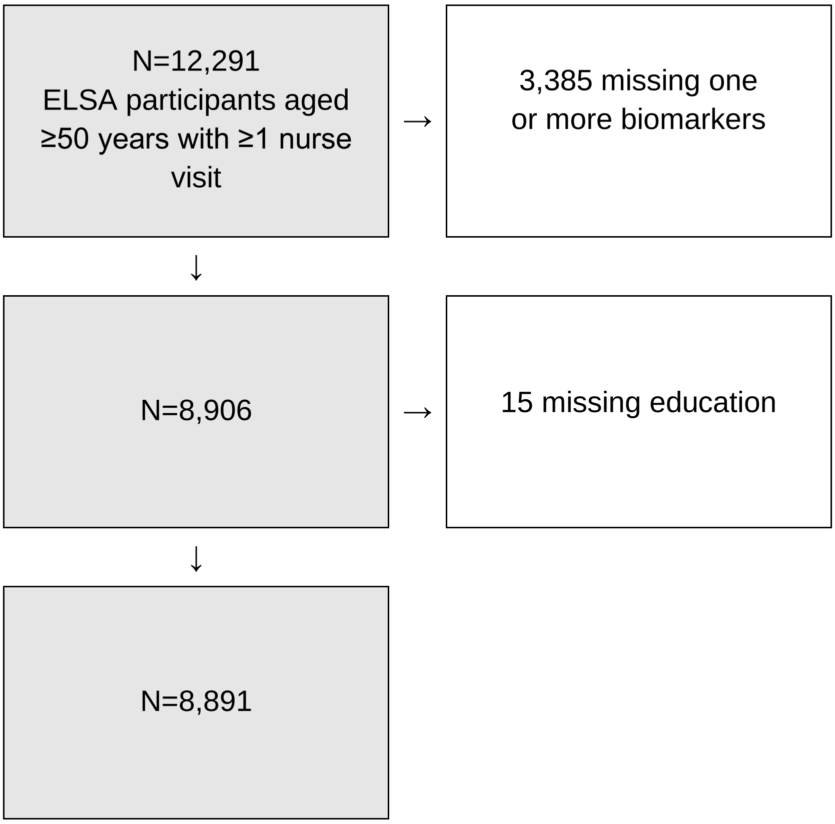

Supplement: AA-24-1386_Appendix_2_afaf067 [file aa-24-1386_appendix_2_afaf067.pdf]
