# Supplementary material for: Sex and education differences in trajectories of physiological ageing: longitudinal analysis of a prospective English cohort study
Source: Age Ageing. 2025 Mar 29;54(4):afaf067. doi: 10.1093/ageing/afaf067 (PMC11954548; doi:10.1093/ageing/afaf067)
Supplement: AA-24-1386_Appendix_3_afaf067 [file aa-24-1386_appendix_3_afaf067.pdf]

Sex and education differences in trajectories of physiological ageing: longitudinal analysis of a prospective English cohort study

Appendix 3. Supplemental tables

Table 3a. Weighting of biomarkers included in physiological score.

Biomarker	Weight
Pulse pressure	0.42
Systolic blood pressure	0.40
Fibrinogen	0.23
C-reactive protein	0.22
Glycated haemoglobin	0.16
FEV	0.47
FVC	0.47
Grip strength	0.31

Abbreviations: FVC, forced vital capacity; FEV, forced expiratory volume in one second. Weighting is based on principal component analysis described in Methods 1b.

Table 3b. Correlation between physiological age and chronological age at each wave of physiological age estimation.

Wave	Correlation coefficient
2	0.78
4	0.76
6	0.77

Table 3c. Characteristics of participants excluded due to missing data.

	**In analytic sample** N=8,891	**Excluded** N=3,400
Sex		
Male	4094 (46.1)	1495 (44.0)
Female	4797 (53.9)	1905 (56.0)
Age, mean (SD)	63.4 (9.1)	66.3 (10.9)
Education level		
Less than high school	3227 (36.3)	1617 (47.5)
High school diploma	4149 (46.7)	1333 (39.2)
Above high school	1515 (17.0)	427 (12.6)
Data not available	0 (0.0)	23 (0.7)

Data are N (%) unless otherwise indicated.
Abbreviations: SD, standard deviation.

Table 3d. Coefficients for interaction terms from additional analyses.

Model	Coefficient (95% CI)	P-value
(1) Sex x birth cohort (ref: male)		
Female x birth cohort	-0.2 (-1.1 to 0.6)	0.61
Female x birth cohort x CA	0.0 (-0.0 to 0.4)	0.28
(2) Education x birth cohort (ref. low)		
Intermediate x birth cohort	0.4 (-0.5 to 1.3)	0.37
High x birth cohort	0.1 (-1.2 to 1.5)	0.85
Intermediate x birth cohort x CA	-0.0 (-0.1 to 0.0)	0.095
High x birth cohort x CA	-0.0 (-0.1 to 0.0)	0.32

Unit for chronological age and physiological age is years and models are centred at chronological age 50. Where ‘x’ denotes an interaction, Model 1 also includes sex, birth cohort, CA, sex x CA, and birth cohort x CA and Model 2 also includes sex, birth cohort, education, CA, sex x CA, birth cohort x CA, and education x CA.

Birth cohort is fitted as a continuous variable (seven-year birth cohorts from 1911-1962) centred at the 1939-45 cohort; interpretation of interaction coefficients is therefore, for example, the change in sex difference per seven-year increase in birth year (sex x birth cohort), or the change in the sex difference in rate of physiological ageing per seven-year increase in birth year (sex x birth cohort x CA).

Abbreviations: CA, chronological age.
